# Comparative deep transcriptional profiling of four developing oilseeds

**DOI:** 10.1111/j.1365-313X.2011.04751.x

**Published:** 2011-10-10

**Authors:** Manuel A Troncoso-Ponce, Aruna Kilaru, Xia Cao, Timothy P Durrett, Jilian Fan, Jacob K Jensen, Nick A Thrower, Markus Pauly, Curtis Wilkerson, John B Ohlrogge

**Affiliations:** 1 Department of Plant Biology, Michigan State UniversityEast Lansing, MI 48824, USA; 2 Great Lakes Bioenergy Research Center, Michigan State UniversityEast Lansing, MI 48824, USA; 3 MSU-DOE Plant Research Laboratory, Michigan State UniversityEast Lansing, MI 48824, USA; 4 Department of Biochemistry and Molecular Biology, Michigan State UniversityEast Lansing, MI 48824, USA

**Keywords:** lipid metabolism, triacylglycerol synthesis, fatty acid biosynthesis, pyrosequencing, expressed sequence tags, comparative transcriptomics

## Abstract

Transcriptome analysis based on deep expressed sequence tag (EST) sequencing allows quantitative comparisons of gene expression across multiple species. Using pyrosequencing, we generated over 7 million ESTs from four stages of developing seeds of *Ricinus communis*, *Brassica napus*, *Euonymus alatus* and *Tropaeolum majus*, which differ in their storage tissue for oil, their ability to photosynthesize and in the structure and content of their triacylglycerols (TAG). The larger number of ESTs in these 16 datasets provided reliable estimates of the expression of acyltransferases and other enzymes expressed at low levels. Analysis of EST levels from these oilseeds revealed both conserved and distinct species-specific expression patterns for genes involved in the synthesis of glycerolipids and their precursors. Independent of the species and tissue type, ESTs for core fatty acid synthesis enzymes maintained a conserved stoichiometry and a strong correlation in temporal profiles throughout seed development. However, ESTs associated with non-plastid enzymes of oil biosynthesis displayed dissimilar temporal patterns indicative of different regulation. The EST levels for several genes potentially involved in accumulation of unusual TAG structures were distinct. Comparison of expression of members from multi-gene families allowed the identification of specific isoforms with conserved function in oil biosynthesis. In all four oilseeds, ESTs for Rubisco were present, suggesting its possible role in carbon metabolism, irrespective of light availability. Together, these data provide a resource for use in comparative and functional genomics of diverse oilseeds. Expression data for more than 350 genes encoding enzymes and proteins involved in lipid metabolism are available at the ‘ARALIP’ website (http://aralip.plantbiology.msu.edu/).

## Introduction

Seeds store oil in the form of triacylglycerol (TAG) to provide carbon and energy reserves that support establishment of the seedling after germination. These oils are also a major food for humans and are increasingly used for non-food applications. A variety of crops, including soybean, rapeseed, and sunflower produce 20–50% of dry weight (DW) oil in their seeds. World production from oilseed crops was approximately 100 billion kg of oil in 2010 with a value near US$140 billion. Vegetable oil consumption is expected to almost double by 2030 (USDA 2011). Better understanding of lipid biosynthesis and its regulation in both model and non-model plants is likely to be one key to meet this demand as well as to improve the content and composition of oils used for food or other applications.

Within the species that accumulate oil as a major seed storage reserve, substantial diversity is observed in TAG structure, rate of oil synthesis, level of accumulation, and whether oil is stored in the embryo or endosperm tissue. Despite extensive studies for more than 30 years, a number of molecular and biochemical factors associated with these variations among oilseeds remain poorly understood. To develop insight into conserved and diverse aspects of lipid metabolism across multiple species, it is useful to expand the genomic and transcriptomic resources available for non-model species to allow comparative analyses.

The identification of several hundred genes involved in lipid biosynthesis has been facilitated by extensive annotation of the Arabidopsis genome (Li-Beisson *et al.*, 2010; Wallis and Browse, 2010). Their transcription patterns during seed development have been studied using microarrays and conventional expressed sequence tag (EST) sequencing (White *et al.*, 2000; Le *et al.*, 2010; North *et al.*, 2010). Transcription information, in most cases based on conventional EST sequencing and/or microarrays, is also available for developing seeds of *Brassica napus* (Li *et al.*, 2006; Huang *et al.*, 2010), soybean (Vodkin *et al.*, 2004; Jones *et al.*, 2010), *R. communis* (Chen *et al.*, 2007; Lu *et al.*, 2007; Cagliari *et al.*, 2010), flax (Venglat *et al.*, 2011) and other species.

The use of massively parallel pyrosequencing of Arabidopsis can yield increased coverage of genes and more quantitative representation of transcripts compared with traditional DNA sequencing and microarrays (Weber *et al.*, 2007). Compared with other high-throughput methods, pyrosequencing provides longer sequences that aid in assembly and annotation when extensive genomic resources are not available. In addition, large EST datasets are particularly valuable for quantitative and cross-species comparisons of gene expression, whereas microarray data may be influenced by inter-platform variability and nonspecific cross-hybridization (Kothapalli *et al.*, 2002).

For this study we have generated more than 7 million ESTs at four stages of seed development and for four different oilseeds: rapeseed (*Brassica napus*), castor (*Ricinus communis*), burning bush (*Euonymus alatus*), and nasturtium (*Tropaeolum majus*). The goal of this study was to generate a comprehensive EST resource and conduct comparative transcriptome analysis of genes involved in fatty acid (FA) synthesis, TAG accumulation and provision of precursors for these pathways. Analysis of the 16 datasets revealed highly conserved patterns of co-regulation of pathways in the four species and provided insights into seed carbohydrate and lipid metabolism. The large number of ESTs has also allowed us to more accurately assess the expression of less abundant transcripts. In addition to similarities and distinct expression patterns for the four developing oilseeds, we present additional findings enabled by in-depth coverage of the transcriptome, including identification of specific isoforms within gene families that are involved in oil biosynthesis and specialized features associated with unusual oil structures.

## Results and Discussion

**Figure 1:**
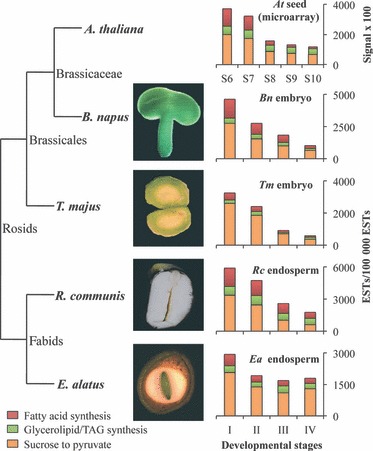
Summary of temporal patterns of expressed sequence tags (ESTs) for oilseeds. The NCBI taxonomy database was used to generate a cladogram of these species that store oil primarily in either embryo or endosperm. Histograms present the sum of ESTs for fatty acid (FA) synthesis and glycerolipid/TAG synthesis, and sucrose to pyruvate pathways, compared between the four species and across the developmental stages. Microarray data (Schmid *et al.*, 2005) for stage 6–10 (mid-late torpedo embryo to green cotyledons) developing seeds of Arabidopsis are included for comparison (data for earlier stages included silique tissue and are not comparable).

The four oilseed species selected for comparative transcriptional profiling exhibit distinct characteristics and phylogenic relationships (Figure 1 and Table 1). The two members of Brassicales, *B. napus* and *T. majus* store oil predominantly in embryos but differ in their oil content (45 and 10% DW, respectively). In contrast, the members of the fabids, *R. communis* and *E. alatus*, store TAGs primarily in the endosperm rather than the embryo. Both species accumulate a similar high oil content (60 and 50% DW, respectively), and both produce TAGs with an unusual structure. In *R. communis* >90% of the acyl chains of the TAGs are in the form of the hydroxy FA, ricinoleic acid. The endosperm of *E. alatus* produces 95% of its oil as acetyl-TAGs (acTAGs), where the *sn*-3 position of glycerol is esterified with acetate rather than long-chain FAs.

**Table 1:** Details of four oilseeds selected for transcriptional profiling

EST analysis	*Brassica napus*	*Tropaeolum majus*	*Ricinus communis*	*Euonymus alatus*
Tissues analyzed	Embryo	Embryo	Endosperm	Endosperm
Developmental stages referred as Stage I–IV	14–20, 21–25, 26–30, and 31–35 DAF	16, 18, 22, and 25 DPA	Stage III, IV, VI and VII+VIII	Harvested on 22 and 29 Aug., 6 and 19 Sept
% Oil	45%	10%	60%	50%
Sequencing platform	454 Titanium	454 FLX	454 FLX	454 FLX
No. of ESTs (million)	2.3	1.5	0.8	1.7
No. of Arabidopsis orthologs	17 405	14 983	12 788	15 343
% Of ESTs related to lipid biosynthesis	1.1	0.4	1.9	0.6
% Of ESTs related to sucrose to pyruvate synthesis	1.5	1.4	1.9	1.5

EST, expressed sequence tag; DAF, days after flowering; DPA, days post-antithesis.

Four stages during embryo development of *B. napus* and *T. majus* and endosperm of *R. communis* and *E. alatus*, beginning at the period when oil synthesis was rapidly increasing, were chosen for transcriptome analysis. In total, cDNA libraries for 16 tissue samples from the four species were subjected to pyrosequencing and 7 million ESTs (200–400 nucleotides in length) were generated (Table 1). In order to provide a common reference to compare EST assemblies from the four species, individual contigs were annotated based on the highest BLASTX score against the Arabidopsis proteome (*E*-value cut-off <10^−10^). Among higher plants, the Arabidopsis proteome is the most completely annotated and experimentally verified, and includes a recent update of information on several hundred genes involved in lipid metabolism (Li-Beisson *et al.*, 2010; http://aralip.plantbiology.msu.edu/). Orthologs of ∼13 000–17 000 Arabidopsis proteins were represented in the four oilseeds (Table 1). The EST counts assigned to each gene were expressed as ESTs/100 000 ESTs and this value was used as a measure to compare relative gene expression across species. The results presented below (and in Table S1) focus on proteins involved in seed metabolic pathways that convert sucrose to TAG. In addition, complete datasets for orthologs represented by ≥10 ESTs are presented in Table S5.

To provide a general overview of the transcriptional patterns for each oilseed, the ESTs related to lipid and carbohydrate metabolism were broadly divided into three functional categories based on their roles in metabolism (Table S1). The relative proportion of ESTs involved in conversion of (i) sucrose to pyruvate, (ii) plastidial FA synthesis from pyruvate, and (iii) TAG and membrane lipid assembly are shown in Figure 1 at four developmental stages. The ESTs associated with lipid metabolism ranged from 1.9% of the total for *R. communis* (60% oil) to 0.4% for *T. majus* (10% oil), whereas sucrose to pyruvate metabolism was represented by approximately the same percentage (1.4–1.9%) of ESTs across species (Table 1). In all four species, the ESTs associated with oil biosynthesis and sucrose to pyruvate metabolism declined during development (Figure 1).

The decline in relative abundance of ESTs for many oil biosynthesis and glycolytic enzymes during seed development was initially unexpected, because the first developmental stage of the analyzed seeds preceded the major accumulation of oil. A similar decline in the expression of these genes after the torpedo stage of development (<15% of final TAG) was also observed in microarray data from Arabidopsis seeds (Figure 1) and was also noted in proteomic studies of *R. communis* endosperm (Houston *et al.*, 2009). We hypothesize that higher EST levels at the earlier stage may reflect mRNA preceding protein synthesis or the rapid cell division under way and the requisite need for high expression of transcripts for glycolysis and membrane synthesis to support this growth. This EST decline was not observed for other proteins involved in later steps of oil accumulation, and therefore is not a general feature of lipid-related proteins. The ‘bell-shaped’ expression pattern described previously for Arabidopsis transcripts involved in seed FA synthesis was based on samples that included seed coat and endosperm (Ruuska *et al.*, 2004) and siliques (Schmid *et al.*, 2005). As noted previously, these tissues can substantially dilute the mRNA of early stage embryos (Baud and Graham, 2006). In this study, the oil-producing tissues were dissected from the seed coat and other tissues prior to extraction of RNA.

### Transcriptional patterns for most enzymes involved in the conversion of pyruvate to FA were similar in four diverse oilseeds

**Figure 2:**
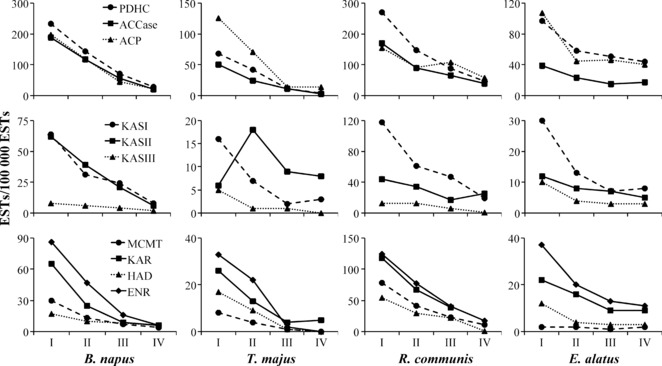
Temporal changes in expressed sequence tag (EST) levels for various fatty acid synthesis enzymes. Temporal changes in EST levels for pyruvate dehydrogenase complex (PDHC), acetyl-CoA carboxylase (ACCase), acyl carrier protein (ACP), ketoacyl-ACP synthase (KASI, -II, and -III), malonyl-CoA:ACP malonyltransferase (MCMT), ketoacyl-ACP reductase (KAR), β-hydroxyacyl-ACP dehydratase (HAD), and enoyl-ACP reductase (ENR), during embryo or endosperm development. The EST levels for PDHC and nuclear-encoded ACCase subunits and also for multiple isoforms within a gene family were summed. For subunit-specific details see Table S1a and Figure S1.

The acetyl-CoA precursor required for *de novo* FA synthesis is provided by the activity of the pyruvate dehydrogenase complex (PDHC). Four enzyme subunits (E1-α and -β, E2, and E3) contribute to PDHC activity. The temporal expression pattern of PDHC followed a similar declining trend in the four oilseeds (Figures 2 and S1, Table S1). The PDHC enzyme occurs in both plastids and mitochondria (Randall *et al.*, 1989). In the three oilseeds producing the most oil, ESTs for plastid PDHC were four to five-fold higher than the mitochondrial PDHC (Table S1a). These data at the transcript level reinforce and extend to other seeds the conclusion based on flux studies of *B. napus* that most acetyl-CoA required for FA synthesis is produced by the plastid PDHC (Schwender *et al.*, 2006). The EST levels for alternative enzymes that might provide acetyl units for FA synthesis, including ATP citrate lyase, acetyl-CoA synthase, or carnitine acetyltransferase, were either substantially lower than PDHC or not detected (Table S5). Thus, the EST data have helped to distinguish between alternative scenarios for plastid acetyl-CoA production in these species.

Carboxylation of acetyl-CoA to malonyl-CoA is the first committed step in FA synthesis and is catalyzed by a multi-subunit acetyl-CoA carboxylase (ACCase) complex. Among the three nuclear-encoded subunits of the heteromeric ACCase enzyme, α**-**carboxyltransferase (CT) ESTs followed by biotin carboxylase (BC) were most abundant in *B. napus*, *R. communis* and *E. alatus* seeds (Figure 2). The ESTs for the three ACCase subunits displayed a coordinated temporal pattern (Figure S1). These data confirm earlier microarray and northern blot data for Arabidopsis, which indicate a constant ACCase subunit stoichiometry during seed development (Ke *et al.*, 2000; Baud and Lepiniec, 2009). With the exception of Arabidopsis and *B. napus*, where two isoforms of biotin carboxyl carrier protein (BCCP) are expressed in seeds (Thelen *et al.*, 2001), expression of multiple BCCP isoforms in the other oilseeds has not been specifically examined. Expressed sequence tag levels for orthologs of BCCP2 were more abundant than BCCP1 in seeds of *R. communis*, *B. napus*, and *T. majus*. In contrast, BCCP1 was the only isoform detected in *E. alatus*. A second form of ACCase with a homodimeric structure occurs in *B. napus* plastids (Schulte *et al.*, 1997). The ESTs for this ACCase were either undetectable or 3–40-fold lower than the multi-subunit ACCase (Table S1a); therefore, its role in plastid metabolism remains enigmatic.

Plastidial acetyl-CoA and malonyl-CoA are converted into long-chain acyl-ACP (acyl carrier protein) by a series of reactions involving seven enzymes with ACP as a cofactor. The temporal profiles for ACP and the FA synthesis enzymes were very similar to that of PDHC and ACCase (Figure 2 and Table S1a). Eighteen carbon acyl-ACP products generated by FA synthase undergo desaturation by stearoyl-ACP desaturases (SAD). The EST levels for SAD were more abundant than for any other enzyme involved in FA synthesis (Table S1a) consistent with the low catalytic efficiency of SAD (Shanklin and Cahoon, 1998). In Arabidopsis, there are seven isoforms of SAD (Shanklin *et al.*, 2009) of which At2g43710 is the most highly expressed in seeds. Of note, orthologs of At2g43710 were also the most abundant in the four species (Table S1a), implying an evolutionarily conserved and likely distinctive function for this isoform.

The timing and ratio of expression of transcripts that encode the two acyl-ACP thioesterases that terminate plastid FA synthesis, FATA and FATB (Voelker, 1996), did not follow the pattern of the other FA synthesis enzymes. In particular, EST levels for FATB (responsible for most saturated FA production) did not decline during development (except for *T. majus*). In general FATA ESTs were higher than FATB, consistent with greater plastid production of unsaturated than saturated FAs in these seeds. In *R. communis* FATA EST levels were 1000-fold higher than FATB (Figure S1, Table S1a), a ratio that may explain the very low saturated FA content (∼2%) of *R. communis* seeds.

The free FAs generated by thioesterases in the plastid are esterified to CoA by long-chain acyl-CoA synthetases (LACS) at the plastid envelope. There are nine LACS isoforms in Arabidopsis (Fulda *et al.*, 2002; Shockey *et al.*, 2002; Schnurr *et al.*, 2004). Among these AtLACS9 (At1g77590) is seed-expressed and plastidial and ESTs for LACS9 orthologs were the most abundant isoform in all four oilseeds (Table S1a). These data imply an evolutionarily conserved role for LACS9 as the major LACS isoform associated with export of FAs from seed plastids.

### Conserved stoichiometry and temporal regulation of plastid FA synthesis

**Figure 3:**
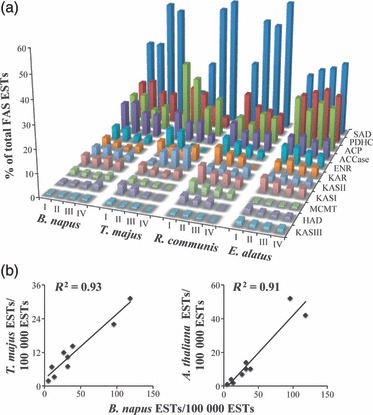
Conserved stoichiometry and correlation in expressed sequence tag (EST) levels for genes encoding pyruvate dehydrogenase complex (PDHC), acyl carrier protein (ACP) and FA synthesis (FAS) enzymes. (a) The percentage of ESTs for PDHC, ACP, and FA synthesis enzymes relative to the sum of ESTs for the pathway are shown. (b) The EST levels for *B. napus* FA synthesis enzymes (PDHC, acetyl-CoA carboxylase, malonyl-CoA:ACP malonyltransferase, ketoacyl-ACP synthase I, II, and III, ketoacyl-ACP reductase, β-hydroxyacyl-ACP dehydratase, and enoyl-ACP reductase) were strongly correlated with that of *T. majus* and with *Arabidopsis thaliana* 7-day seedlings.

The availability of 16 datasets allowed us to examine the extent to which the stoichiometry of FA synthesis gene expression during development is conserved in the four species. To evaluate stoichiometry, we calculated the ratio of ESTs for each of nine core FA synthesis enzymes, ACP, and PDHC to the sum of ESTs for the same proteins. Interestingly, the EST levels for these proteins displayed a similar stoichiometry between species and during seed development (Figure 3a). For example, in all cases, ESTs for ketoacyl-ACP synthase III (KASIII) were the lowest whereas those for SAD were the most abundant (Figure 3a). The stoichiometry of SAD and ACP ESTs was the most variable, but as shown in Figure 3b, direct comparison of EST levels for the enzymes PDHC, ACCase, malonyl-CoA:ACP malonyltransferase (MCMT), KASI, -II, -III, ketoacyl-ACP reductase (KAR), hydroxyacyl-ACP dehydratase (HAD), and enoyl-ACP reductase (ENR) indicated a tight correlation (*R*^2^ = 0.93) between *B. napus* and *T. majus* ESTs and *R*^2^ ≥ 0.81 for all other species to species comparisons (Table S2a).

In addition to the conserved stoichiometry *between* species, most comparisons of temporal expression *within* species were highly correlated between the four stages of development (*R*^2^ ≥ 0.9 for *B. napus*; Table S2b). In Arabidopsis seeds, gene expression for most FA synthesis proteins is regulated by the WRINKLED1 (WRI1) transcription factor (Cernac and Benning, 2004; Baud *et al.*, 2009; Maeo *et al.*, 2009). The temporal expression of WRI1 orthologs of *B. napus* and *R. communis* closely matched the patterns of its target genes (Tables S1a and S3).

We also asked whether the stoichiometric relationships between the nine enzymes described above for seeds extends to non-seed tissues. When the *B. napus* embryo EST levels were plotted against pyrosequencing EST data for 7-day Arabidopsis seedlings (Weber *et al.*, 2007) a high correlation (*R*^2^ > 0.9; Figure 3b and Table S2a) was again observed (although lower a *R*^2^ with other species). Together, the above comparative expression analyses indicate that controls over FA synthesis transcript expression is highly conserved between four different species and two seed tissues (embryo and endosperm), and also during seed development. Furthermore, the stoichiometric expression of the nine enzymes extends to vegetative tissues, where WRI1 expression is very low. Thus, other factors, in addition to WRI1, can be expected to play a role in maintaining these highly conserved relationships.

### Acyl-CoA to TAG synthesis: acylation of *sn*-1 and *sn*-2, but not *sn*-3, are similar across the four oilseeds

**Figure 4:**
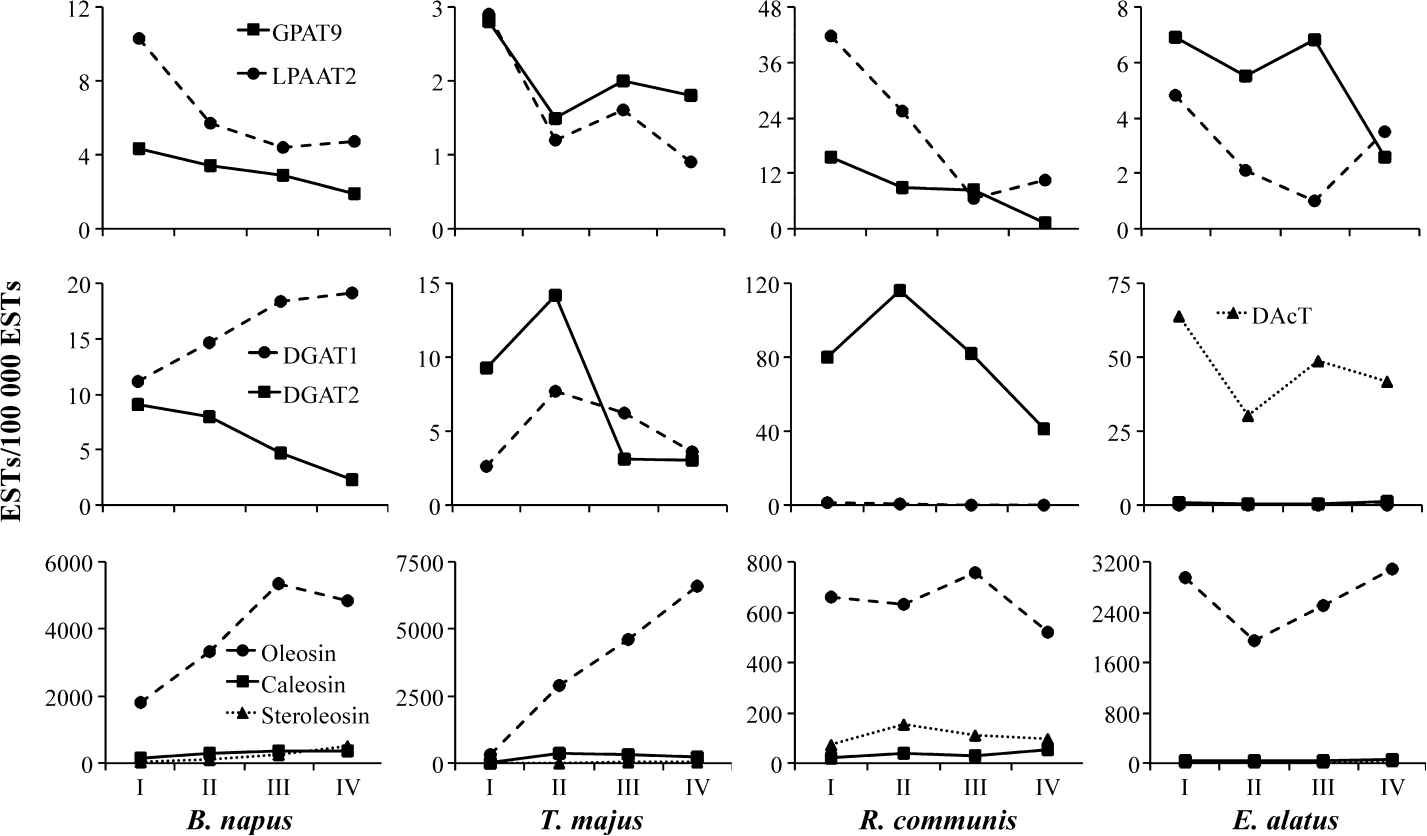
Temporal profile of expressed sequence tags (ESTs) for enzymes involved in acylation of *sn*-1, *sn*-2, and *sn*-3 positions of glycerol-3-phosphate and for oilbody proteins. The EST levels for putative glycerol-3-phosphate acyltransferase (GPAT9), lysophosphatidic acid acyltransferase (LPAAT2), and diacylglycerol acyltransferases (DGAT1, -2, and an acetyl transferase, specific to *E. alatus*, DAcT) are shown. The EST levels for all isoforms of oleosins, caleosins, or steroleosins were summed. For isoform-specific details see Table S1a.

After FAs are synthesized in the plastid, exported and activated to form acyl-CoAs, the acyl chains are assembled into TAG by a series of membrane-associated reactions. Glycerol-3-phosphate acyltransferases (GPAT) catalyze *sn*-1 acylation of glycerol-3-phosphate to yield lysophosphatidic acid (LPA). An eight-member GPAT gene family was identified based on similarity to yeast GPAT sequences (Zheng *et al.*, 2003). Several members of this family are *sn*-2 acyltransferases that are involved in the production of cutin and suberin rather than membrane or storage glycerolipids (Beisson *et al.*, 2007; Yang *et al.*, 2010). The GPAT(s) involved in membrane and storage glycerolipid synthesis in plants have not yet been clearly identified. A candidate for this enzyme, termed AtGPAT9 (At5g60620), possesses little similarity to the GPAT1-8 family but is the Arabidopsis protein most similar to a mammalian GPAT important for TAG synthesis (Cao *et al.*, 2006; Gidda *et al.*, 2009). Expressed sequence tags encoding orthologs of AtGPAT9 were present in the four oilseeds examined (Figure 4), whereas GPAT1–8 were either absent or expressed at low levels (Table S1a). Although a biochemical role for GPAT9 in TAG synthesis remains to be demonstrated, EST data from all four species are consistent with the hypothesis that GPAT9 is an initial acyltransferase for seed glycerolipid assembly.

The second acylation in *de novo* TAG assembly is catalyzed by LPA acyltransferase (LPAAT). Activity of LPAAT has been demonstrated for two of the five Arabidopsis isoforms of this enzyme family. Of these, AtLPAAT2 (At3g57650) is the most highly expressed and is essential for development of the female gametophyte (Kim *et al.*, 2005). Our EST data further demonstrate that orthologs of AtLPAAT2 are the major LPAAT isoform expressed in all four oilseeds (Figure 4, Table S1a) extending the recent characterization of LPAAT isozymes in *B. napus* (Maisonneuve *et al.*, 2010). Thus, for the first two steps of glycerol acylation in oilseeds, the EST data have helped to clarify which of several alternative gene family members are likely to be responsible for TAG biosynthesis in several species.

The final step in TAG biosynthesis is the acylation of diacylglycerol (DAG) to form TAG. Two different classes of enzymes, diacylglycerol acyltransferases (DGAT) and phospholipid:diacylglycerol acyltransferases (PDAT), can catalyze this reaction, using either acyl-CoAs or phospholipids, respectively, as the acyl donor. Two unrelated types of DGAT enzymes have been confirmed to play a role in plants (Cases *et al.*, 1998; Lardizabal *et al.*, 2001). In Arabidopsis, DGAT1 is the predominant enzyme synthesizing TAG in seeds (Katavic *et al.*, 1995; Routaboul *et al.*, 1999; Zou *et al.*, 1999). In *R. communis* and tung, DGAT2 has been proposed to be important for incorporation of unusual FAs into TAG (Kroon *et al.*, 2006; Shockey *et al.*, 2006; Burgal *et al.*, 2008) but DGAT2 is also abundant in olive (Alagna *et al.*, 2009) and oil palm (Bourgis *et al.*, 2011; Tranbarger *et al.*, 2011) that accumulate normal TAG. In *B. napus*, ESTs for DGAT1 are more abundant than ESTs for DGAT2, whereas in *R. communis*, which incorporates hydroxylated acyl chains into TAG, RcDGAT2 is expressed at very high levels (∼80 ESTs/100 000 ESTs) while DGAT1 is essentially absent (Figure 4). In *E. alatus*, a novel acetyltransferase enzyme (EaDAcT) catalyzes the terminal step (i.e. *sn*-3 acetylation) in the synthesis of the acTAGs that comprise 95% of endosperm oil (Durrett *et al.*, 2010). Intriguingly, although DGAT1 and DGAT2 were expressed in other *E. alatus* tissues that produce normal TAG, their ESTs were undetectable in *E. alatus* endosperm (Figure 4; Durrett *et al.*, 2010). The final step in TAG synthesis can also be catalyzed by PDATs, which transfer the *sn*-2 acyl group from phospholipids to DAG (Dahlqvist *et al.*, 2000). The EST levels of PDAT1 and PDAT-like/PDAT2 (At3g44830) orthologs in these oilseeds (Figure 7 and Table S1a) were consistently much lower compared with the levels of the different DGAT ESTs. As with DGAT1 and DGAT2, ESTs for a PDAT1 ortholog were undetectable in *E. alatus* endosperm.

In addition to the enzymes presented above, other acyltransferases play an important role in TAG biosynthesis in oilseeds. For example, in developing soy embryos, about 60% of newly synthesized FAs are first incorporated into the *sn*-2 position of phosphatidylcholine (PC) rather than onto glycerol-3-phosphate (Bates *et al.*, 2009). Lysophosphatidylcholine acyltransferases (LPCATs) or related enzymes possibly involved in such reactions remain to be identified. Expressed sequence tags for orthologs of At1g12640, which was shown to have LPCAT activity *in vitro* (Stahl *et al.*, 2008), were much higher in *R. communis* relative to *B. napus* (Figure 7) and the two other oilseeds. It is interesting to note that the temporal expression profile of this *R. communis* candidate LPCAT is similar to that of RcDGAT2 involved in TAG biosynthesis, suggesting possible co-regulation and involvement in the same biochemical pathway. Expressed sequence tags for orthologs of another uncharacterized PDAT-related gene (At4g19860) were present at similar or higher levels than LPCAT in the other oilseeds, suggesting that further study of a possible role in TAG biosynthesis may be useful (Table S1a).

**Figure 5:**
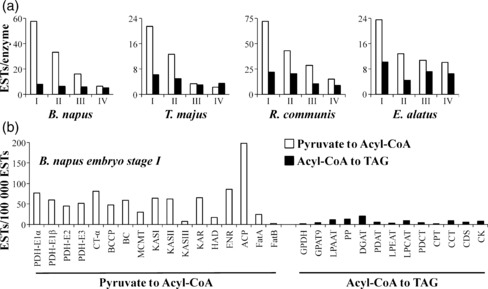
Comparison of expressed sequence tag (EST) levels for enzymes involved in plastidial and non-plastidial reactions of triacylglycerol (TAG) biosynthesis. (a) The EST levels per enzyme for reactions from pyruvate to acyl-CoA declined during seed development but were higher than non-plastidial reactions. (b) The EST levels for each enzyme are indicated for stage I of *B. napus* embryo. The ESTs for multiple isoforms within a gene family were summed. Desaturases are not shown.

Comparison of EST data for the above non-plastid enzymes involved in TAG assembly with the ESTs for enzymes of plastid FA synthesis revealed two noteworthy differences. First, EST levels of the individual FA synthesis enzymes were on average five-fold higher than the EST levels for glycerolipid and TAG assembly enzymes (Figure 5a,b; stage I data). Second, the expression of the genes encoding FA synthesis enzymes decreased on average five-fold from stage I to IV (Figure 5a). In contrast, most ESTs associated with TAG assembly were either constant or slightly decreased; DGAT was one notable exception which increased (Table S1a). These comparisons strongly suggest that regulation of transcripts for the plastidial and non-plastidial reactions of oil synthesis are under separate controls.

### Storage of TAG

Triacylglycerols accumulate in oilbodies, which consist of a TAG core surrounded by a phospholipid monolayer and abundant amphipathic proteins. Three classes of proteins, oleosins, steroleosins, and caleosins, are associated with seed oilbodies (Naested *et al.*, 2000; Lin *et al.*, 2002; Jolivet *et al.*, 2004). The EST levels for the oilbody proteins increased or remained high during development, a pattern quite distinct from that of FA synthesis. The ESTs for oleosins (Jolivet *et al.*, 2004) were from 13- to more than 100-fold higher than ESTs for steroleosins or caleosins (except for *R. communis*; Figure 4). These data also provide insight into which isoforms of these large gene families encoding oleosins and caleosins are candidates for further characterization. In case of steroleosins, orthologs of only two of eight Arabidopsis genes (At5g50700 and At4g10020) were detected (Table S1a).

### Expression patterns associated with the synthesis of unusual TAGs

**Figure 6:**
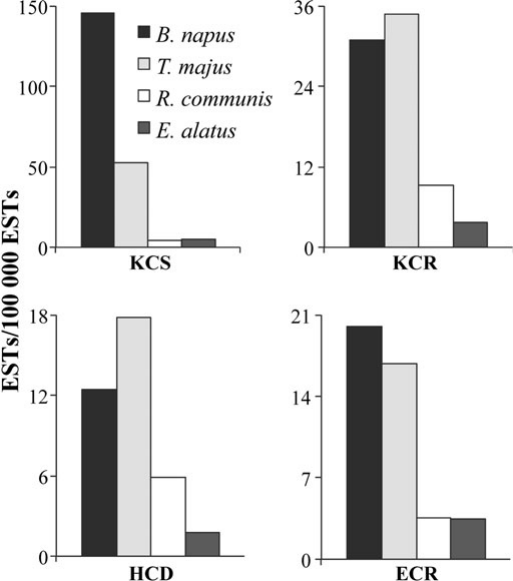
Species-specific expression of four enzymes of the very long-chain fatty acid (VLCFA) synthesis pathway. The expressed sequence tag (EST) levels (average stage I and II) for genes encoding ketoacyl-CoA synthase (KCS), ketoacyl-CoA reductase (KCR), hydroxyacyl-CoA dehydratase (HCD), and enoyl-CoA reductase (ECR). The ESTs for multiple isoforms were summed. For isoform-specific annotations see ‘fatty acid elongation’, Table S1a.

In contrast to the 16–18 carbon FA of *R. communis* and *E. alatus*, the storage oils of *T. majus* and *B. napus* are characterized by a high (>50%) proportion of very long chain fatty acids (VLCFA). Erucic acid (C22:1), the most abundant VLCFA is synthesized from oleoyl-CoA by elongation catalyzed by ketoacyl-CoA synthase (KCS), ketoacyl-CoA reductase (KCR1), hydroxyacyl-CoA dehydratase (HCD), and enoyl-CoA reductase (ECR). The ESTs for all four enzymes were several-fold higher in *T. majus* and *B. napus* in comparison to *R. communis* or *E. alatus.* (Figure 6), Thus, erucic acid biosynthesis is clearly associated with higher expression of transcripts for all four enzymes of the pathway. *Brassica napus* ESTs for the cytosolic homomeric ACCase that provides malonyl-CoA for elongation were 40-fold greater than for *E. alatus* or *R. communis*. Interestingly, the loss of KCS function (Roscoe *et al.*, 2001) in the *B. napus* cultivar studied here appears to have caused no compensatory or feedback reduction in expression of the other enzymes of the elongation pathway or of ACCase.

**Figure 7:**
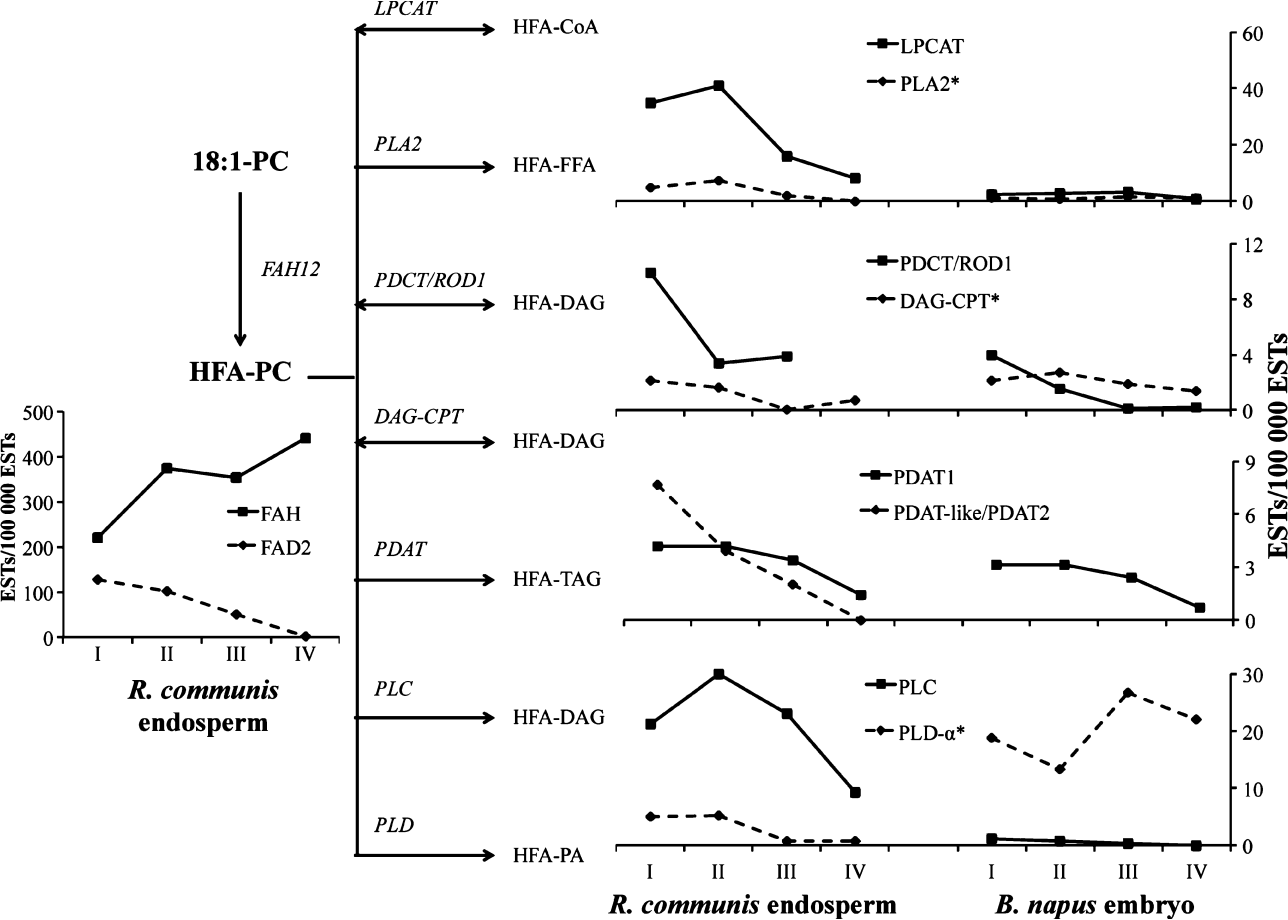
Transcriptional profile of enzymes potentially involved in hydroxy fatty acid (HFA) accumulation in *R. communis*. Expression levels of fatty acid hydroxylase (FAH) are compared with its close homolog, fatty acid desaturase (FAD2). The expressed sequence tag (EST) levels for enzymes potentially involved in removal of ricinoleate from phosphatidylcholine (PC) are compared between *R. communis* and *B. napus*. Asterisk indicates that EST levels for members of the same gene-family were summed. LPCAT, lysophosphatidylcholine acyltransferase; PLA2, phospholipase A2; PDCT/ROD1, phosphatidylcholine:diacylglycerol cholinephosphotransferase; DAG-CPT, diacylglycerol cholinephosphotransferase; PDAT, phospholipid/glycerol acyltransferase; PLC, phospholipase C; PLD, phospholipase D; FFA, free fatty acid; DAG, diacylglycerol; TAG, triacylglycerol; PA, phosphatidic acid.

In *R. communis*, ricinoleic acid is synthesized by a fatty acid hydroxylase (FAH) very closely related in sequence to the FAD2 desaturase. Fatty acid hydroxylases introduce a hydroxyl group to oleate at the *sn*-2 position of PC (van de Loo *et al.*, 1995). In *R. communis*, the expression of *FAH* is not only much higher than the *FAD2* ortholog, but increases during endosperm development, whereas the FAD2 ortholog decreases (Figure 7). The expression of *FAH* in *R. communis* is also several fold higher than *FAD2* expression in the other oilseeds, and this is perhaps required to achieve the very high (>90%) hydroxy FA content of TAG in *R. communis*.

Very little ricinoleate accumulates in PC, indicating its rapid removal after synthesis. The enzymatic steps responsible for this movement are not well defined. A summary illustrating seven alternative routes that could direct ricinoleate from PC to TAG is provided in Figure 7 with comparison of ESTs between *R. communis* and *B. napus*. In addition to much higher LPCAT ESTs in *R. communis* noted above, ESTs for orthologs of phospholipase C (PLC) and PDAT-like/PDAT2 (At3g44830) were >10 fold higher in *R. communis* than *B. napus*. In contrast, EST levels of orthologs of choline phosphotransferase (CPT), phospholipase D, PDCT, and phospholipase A did not differ as greatly between *R. communis* and the other oilseeds. Thus, these comparative analyses suggest that orthologs of LPCAT, PDAT-like/PDAT2 and PLC are possible candidates associated with high accumulation of ricinoleate in TAG and its exclusion from membrane lipids. A *R. communis* ortholog of At3g44830 was recently expressed in Arabidopsis expressing FAH but did not increase ricinoleic levels (van Erp *et al.*, 2011). It is possible that combinations of these genes will be needed to achieve very high levels of ricinoleate in transgenic seeds.

### Expressed sequence tag data can distinguish gene family members associated with oil biosynthesis

**Figure 8:**
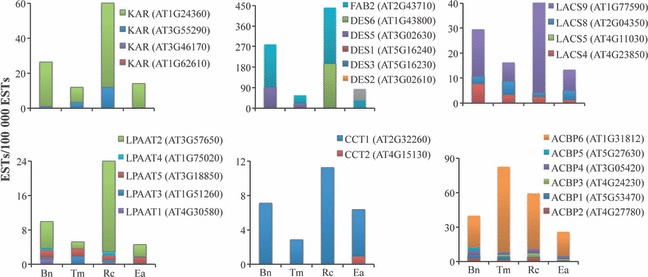
Expression of isoforms of selected multi-gene families involved in lipid biosynthesis. acyl-CoA binding protein (ACBP); long-chain acyl-CoA synthetase (LACS); ketoacyl-ACP reductase (KAR); lysophosphatidic acid acyltransferases (LPAAT); choline-phosphate cytidylyltransferase (CCT); stearoyl-ACP desaturases (SAD/DES).

For enzymes and proteins involved in lipid synthesis that are encoded by more than one gene, it has not been discerned whether these isoforms are functionally redundant or if a specific isoform is involved in seed oil synthesis. For several gene families, comparison of relative expression levels of isoforms across the four species allowed us to identify the most abundantly and consistently expressed isoform(s) involved in a particular reaction. As noted above, for the SAD, LACS, and LPAAT families, the same isoform predominated in all four species. Similar identifications were made for a number of other gene families (Figure 8). Of the two genes that encode DAG-CPT in Arabidopsis, ESTs for orthologs of At1g15360 were several-fold higher than for At3g25585 in each species. Similarly, two genes encode choline-phosphate cytidylyltransferase (CCT) in Arabidopsis (Inatsugi *et al.*, 2009) and orthologs of At2g32260 greatly predominant over At4g15130 in all species (Figure 8). In Arabidopsis, six acyl-CoA binding proteins (ACBPs) have been identified (Xiao and Chye, 2009). Of these, the soluble ACBP6 ortholog was the most predominant transcript in all four oilseeds (Figure 8). Also, there are four candidates for KAR in Arabidopsis. Orthologs of only At1g24360 were highly expressed in all four oilseeds (Figure 8) suggesting that the other three candidates are less likely to participate in seed oil biosynthesis. These examples of evolutionarily conserved isoform expression patterns provide additional levels of annotation and clues to understanding the multi-gene families.

### Providing carbon for FA synthesis: sucrose to pyruvate

**Figure 9:**
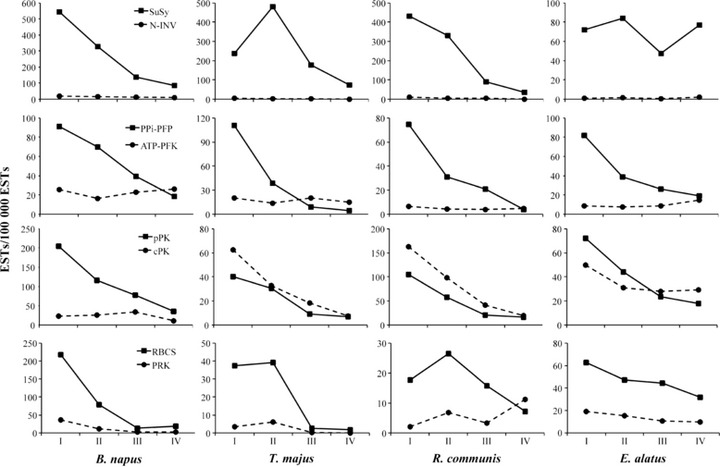
Temporal profile of expressed sequence tags (ESTs) for genes encoding enzymes involved in the conversion of sucrose to pyruvate. The EST levels for sucrose synthase (SuSy), neutral invertase (N-INV), pyrophosphate-dependent phosphofructokinase (PPi-PFP), ATP-dependent phosphofructokinase (ATP-PFK), pyruvate kinase (PK), Rubisco small subunit (RBCS), and phosphorubulokinase (PRK) during four stages of oilseed development are shown. The EST data for the cytosolic (c) or plastidial (p) isoform of the enzyme are indicated by a prefix. The ESTs for subunits of an enzyme (pPK, PPi-PFP etc.) and for multiple isoforms of multi-gene families were summed. Annotation and EST levels for each isoform are provided in Table S1b.

Sucrose and glucose are the major source of carbon provided by maternal tissues to developing seeds. In embryos and endosperm of four oilseeds, sucrose synthase (SuSy) ESTs were 20–40-fold higher than neutral invertases (N-INV; Figure 9), implicating SuSy as the preferred enzyme for initial sucrose metabolism. These EST data reinforce and extend enzyme assays of *B.* *napus* (Hill *et al.*, 2003; Morley-Smith *et al.*, 2008) which suggest that SuSy is the major enzyme responsible for generation of hexoses during oil accumulation. Also consistent with this conclusion, fructokinase (FK) ESTs were higher than hexokinase (HXK; Table S1b).

The EST profiles for alternative reactions of fructose-6-phosphate were distinct. Phosphorylation of fructose-6-phosphate to fructose-1,6-bisphosphate is catalyzed irreversibly by ATP-dependent phosphofructokinase (ATP-PFK) and reversibly by pyrophosphate-dependent phosphofructokinase [PPi-PFP; (Plaxton, 1996)]. In all four oilseeds, EST levels of PPi-PFP (only present in cytosol) were much higher (4- to 40-fold) than either cytosolic ATP-PFK or plastid ATP-PFK (Figure 9). This greater abundance of PPi-PFP ESTs together with the high SuSy expression, which generates the substrate for PPi-dependent UDPglucose pyrophosphorylase, emphasizes the importance of PPi as a key metabolite during seed development. Preference for PPi-dependency over ATP-dependency was proposed as a strategy for efficient conservation of oxygen in embryos (Baud and Graham, 2006).

Pyruvate is most directly generated via activity of pyruvate kinase (PK), which occurs in both cytosol (cPK) and plastids (pPK). Interestingly, ESTs for pPK were nine-fold higher than for cPK at stage I of *B. napus* whereas in the other oilseeds the distribution was more balanced between the two compartments (Figure 9). These data reinforce a *B. napus* flux model which indicates that most pyruvate for FA synthesis is generated from phosphoenolpyruvate (PEP) in the plastid (Schwender *et al.*, 2003) and also the observation that oil content of Arabidopsis seeds is reduced 60% in a mutant with reduced plastid PK activity (Andre *et al.*, 2007).

The green embryos of *B. napus* convert a major portion of imported carbohydrate to precursors of FA synthesis through an alternative to glycolysis referred to as the ‘Rubisco bypass’ (Schwender *et al.*, 2004). In this pathway, the activity of Rubisco and phosphoribulokinase (PRK), together with non-oxidative steps of the pentose phosphate pathway, can provide 20% more acetyl-CoA for FA synthesis. The participation of this bypass pathway in *B. napus* was clearly associated with several-fold higher levels of ESTs for Rubisco small subunit (RBCS) and PRK than observed in the other three oilseeds (Figure 9). Intriguingly, although at lower levels, we also observed Rubisco and PRK ESTs in the non-green seeds (Figure 9). Transcripts for both enzymes are not expressed in roots, but occur in EST datasets of Arabidopsis, sesame, *R. communis* and other seeds (http://www.ncbi.nlm.nih.gov/dbEST). A proteomic study identified Rubisco in *R. communis* but at 11-fold lower levels than in *B. napus* (Houston *et al.*, 2009) and Rubisco enzyme activity is comparable to other glycolytic enzymes (Simcox *et al.*, 1977). A conserved role of Rubisco in metabolism in non-green seeds (without light to provide cofactors for ribulose-1,5-bisphosphate generation) warrants further investigation.

### Plastidial and cytosolic glycolysis

Plant glycolysis is compartmentalized, with reactions occurring in both the cytosol and plastid (Dennis and Miernyk, 1982; Plaxton, 1996; Andriotis *et al.*, 2010). The distribution of flux between the two compartments has not been well established. Enzymes for a complete glycolytic pathway in both cytosol and plastid were represented by ESTs from all four seeds. (Table S1b and Figure S2). The ESTs for cytosolic glycolytic enzymes were higher than the plastidial isoforms in every case except PK of *B. napus*. The green *B. napus* embryos were also distinguished from the other three oilseeds by a higher plastid/cytosol ratio for FK, phosphoglucose isomerase (PGI), fructose 1,6-bisphosphate aldolase (FBA), and enolase (ENO; Figure S2). These data suggest that compared with the other species, light reactions in *B. napus* allow a greater proportion of hexose to pyruvate flux in plastids.

### Coordinated expression of carbohydrate and lipid metabolism genes

Self-organizing maps (SOMs) were used to compare temporal patterns of a large number of genes of carbohydrate and lipid metabolism to determine the extent of coordinated expression. We evaluated how temporal expression of 228 genes was distributed into six SOM clusters. Of 39 genes in FA synthesis, 34 (87%) were grouped into two SOM clusters (C1 and C2) with similar declining temporal patterns (Figure S3 and Table S3). Similarly 22 out of 27 plastidial and 24 of 45 cytosolic glycolysis genes (and SuSy) also clustered with the FA synthesis genes. The transcription factors *WRI1*, *LEC1* and *FUS3*, and a majority of plastid transporters were also included. In contrast, *DGAT1* and proteins associated with oilbody formation increased during development (together with *ABI3*) whereas other genes of glycerolipid synthesis were distributed in several temporal clusters. Similar distinctive clustering patterns of FA synthesis and TAG assembly gene expression have recently been described for Arabidopsis seed transcripts (Peng and Weselake, 2011).

## Conclusions

Information on global gene expression during different stages of oilseed development has been largely based on microarray data or on small EST datasets. Comparative transcriptome analysis of multiple, non-model oilseeds has been lacking. In this study, extensive EST datasets have been developed and compared for four oilseeds at four stages of development. In addition to providing new sequence information for genes expressed in diverse oilseeds, the temporal patterns and expression levels for thousands of genes are now available for these species, which can assist future protein/enzyme or other studies.

A theme resulting from analysis of the 16 datasets is that regardless of the oilseed species, or embryo/endosperm storage, ESTs representing almost all reactions of FA synthesis are expressed with comparable stoichiometry and with consistent temporal profiles. Furthermore, the coordinated FA synthesis gene expression patterns extended to many glycolytic and other proteins that provide pyruvate for FA synthesis. These similar transcriptional patterns are likely to be universal aspects of seed development in the plant kingdom. FATB, DGAT, oilbody proteins and RcFAH were among some of the notable exceptions that did not follow the general temporal patterns of FA synthesis ESTs during seed development. In addition, perhaps a surprising observation was that in most cases ESTs for acyltransferases such as GPAT, LPAAT and PDAT that are involved in TAG assembly, did not match the profiles for genes involved in FA synthesis nor did they increase in coordination with DGAT. This suggests that TAG accumulation may not require coordinated expression of acyltransferase transcripts in concert with DGAT and/or may involve post-transcriptional regulation. In addition, these EST analyses were useful in identifying distinctive features pertaining to a specific metabolism; for example, EcDAcT expression in *E. alatus* was accompanied by an absence of DGAT1, DGAT2, and PDAT ESTs and patterns of LPCAT, PLC, and DGAT2 were distinctive in *R. communis*. This study analyzed the expression of only a small subset of the data available for thousands of genes expressed during seed development. We anticipate that other researchers will find the datasets useful for identifying sequences and expression patterns for many other genes expressed in these oilseeds.

## Experimental procedures

### Plant tissue

Seeds of *B. napus* and *R. communis* were collected from greenhouse-grown plants (15 h photoperiod; 22 and 28°C, respectively). *Brassica napus* flowers were tagged on the first day after flowering (DAF) and collected at four developmental stages, 12–20, 21–25, 26–30, and 31–35 DAF. *Ricinus communis* seeds were harvested from stage III to stage VIII, based on embryo length and testa (seed coat) color, as defined anatomically (Greenwood and Bewley, 1982). After removal of the seed coat, *R. communis* seeds were cut longitudinally to separate endosperm and embryo. *Tropaeolum majus* fruits were grown as previously described (Desveaux *et al.*, 1998) and embryos were dissected from seeds collected at 16, 18, 22, and 25 days post-antithesis (DPA). *Euonymus alatus* fruits were collected from the grounds of Michigan State University. Endosperm and embryo tissues were dissected from seeds collected from 22 August to 19 September 2008, times which preceded and included maximal TAG synthesis. For all species, seed coats were removed and RNA was prepared from the main oil storage tissue. All dissected tissues were flash frozen and stored at −80°C for RNA extraction.

### RNA extraction, mRNA purification and cDNA synthesis

Total RNA from frozen tissue of *B. napus* and *R. communis* was extracted using TRIzol reagent (Invitrogen, http://www.invitrogen.com/). The mRNA was purified from 1 mg total RNA with an Illustra mRNA purification kit (GE Healthcare, http://www.gehealthcare.com/) and the quality of the RNA and mRNA was assessed using an Aligent 2100 Bioanalyzer (Agilent, http://www.agilent.com/). *Ricinus communis* cDNA was synthesized using SuperScript Double-Strand cDNA Synthesis Kit (Invitrogen). First-strand cDNA synthesis was performed with 7 μg mRNA and oligo-dT primer (5′ (t)_15_ cga 3′) in 20 μl reaction. Size fractionation of cDNA was performed with CHROMA SPIN +TE-400 columns (Clontech, http://www.clonetech.com/), with quality and size (>0.5 to >6 kb) determined by an Agilent 2100 Bioanalyzer. *Tropaeolum majus* RNA was isolated from embryos as described (Cocuron *et al.*, 2007) and total RNA used for cDNA synthesis. *Brassica napus* and *T. majus* cDNA synthesis was with a Creator SMART cDNA Library Construction Kit (Clontech), using long-distance-PCR. First-strand cDNA was synthesized from 2.0 μg mRNA (*B. napus*) or 1.0 μg total RNA (*T. majus*) using SUPERSCRIPT II reverse transcriptase (Invitrogen), and CDS III/3′ primer (5′tagaggccgaggcggccgacatgttttgtttttttttcttttttttttvn3′). For cDNA amplification by LD PCR, 14 cycles were used for *B. napus* and up to 19 cycles for *T. majus*. After *Sfi*I digestion and size fractionation, cDNA fractions of >500 bp were pooled, precipitated, and resuspended in buffer. The RNA extraction, cDNA library construction, and 454 pyrosequencing for *E. alatus* endosperm and embryo tissues were as described (Durrett *et al.*, 2010).

### DNA sequencing, bioinformatics, and data analysis

Complementary DNA preparations were prepared for sequencing using the Roche Library Preparation Kit (http://www.roche.com/), Roche Emulsion PCR kit and PicoTiterPlates. Sequencing of *B. napus* was performed with the Roche GS FLX Titanium and for other species with Roche GS FLX.

Reads were trimmed to remove low-quality and primer sequences using Seq-Clean and assembled with CAP3 (Huang and Madan, 1999). Initially, 5% of the data were assembled to identify and remove abundant ESTs from the full dataset using BLAT (Kent, 2002). The reduced dataset then underwent two rounds of assembly with CAP3. First-round CAP3 parameter settings for percentage match, overlap length, maximum over-hang percentage, gap penalty, and base quality cut-off for clipping were p90, o50, h15, g2, and c17, respectively. For the second round, overlap length was changed to 100. The resultant contigs were annotated with a translated BLAST against the TAIR8 database (*E*-value cut-off 10^−10^) and further annotated based on information at http://aralip.plantbiology.msu.edu/. The number of ESTs/100 000 ESTs was used as a measure for gene expression. The EST levels and annotations for the oilseed orthologs of >350 Arabidopsis proteins related to lipid and carbohydrate metabolism are provided in Table S1a and S1b. The DNA sequences from this study are deposited at the GenBank Short Read Archive (SRA) with accession numbers provided in Table S4. The EST level data for all orthologs of Arabidopsis proteins (>10 ESTs) are provided in Table S5. Contig nucleotide sequences for *R. communis*, *B. napus* and *E. alatus* are provided as fasta files (RcContigSeq, BnContigSeq and EaContigSeq, respectively).

We used SOMs to evaluate temporal EST expression patterns of 228 proteins related to lipid and carbohydrate metabolism. Expression data were centered and normalized for each protein using adjust methods in Cluster 3.0 (http://bonsai.hgc.jp/~mdehoon/software/cluster/software.htm) and SOM clusters were generated using Gene Cluster 2.0 (http://www.broadinstitute.org/cancer/software/genecluster2/gc2.html).

## SUPPORTING INFORMATION

**Figure S1.** Temporal profile of EST levels for various plastidial proteins.

**Figure S2.** Comparison of average expressed sequence tag levels between cytosolic *versus* plastidial glycolytic enzymes in four oilseeds.

**Figure S3.** Self-organizing maps of temporal expressed sequence tag patterns for 228 B. *napus* proteins of lipid and carbohydrate metabolism.

**Table S1a.** Annotation and expressed sequence tag levels for selected genes associated with lipid metabolism.

**Table S1b.** Annotation and expressed sequence tag levels for selected genes of carbohydrate and organic acid metabolism.

**Table S2a.** Correlation (*R*^2^) of FA synthesis gene expression (average expressed sequence tags for four stages) between the species.

**Table S2b.** Correlation (*R*^2^) of FA synthesis gene expression with time, within each species.

**Table S3.** List of genes included in generating self-organized maps for the developing embryo of *B. napus*.

**Table S4.** Details and accession numbers of expressed sequence tag datasets are available on the NCBI Short Read Archive.

**Table S5a.** Annotation and expressed sequence tag levels for *B. napus* orthologs of Arabidopsis proteins.

**Table S5b.** Annotation and expressed sequence tag levels for *R. communis* orthologs of Arabidopsis proteins.

**Table S5c.** Annotation and expressed sequence tag levels for *E. alatus* orthologs of Arabidopsis proteins.

Please note: As a service to our authors and readers, this journal provides supporting information supplied by the authors. Such materials are peer-reviewed and may be re-organized for online delivery, but are not copy-edited or typeset. Technical support issues arising from supporting information (other than missing files) should be addressed to the authors.

Please note: As a service to our authors and readers, this journal provides supporting information supplied by the authors. Such materials are peer-reviewed and may be re-organized for online delivery, but are not copy-edited or typeset. Technical support issues arising from supporting information (other than missing files) should be addressed to the authors.
