# Supplementary figures and images for: Comparative deep transcriptional profiling of four developing oilseeds
Source: Plant J. 2011 Oct 10;68(6):1014–27. doi: 10.1111/j.1365-313X.2011.04751.x (PMC3507003; doi:10.1111/j.1365-313X.2011.04751.x)

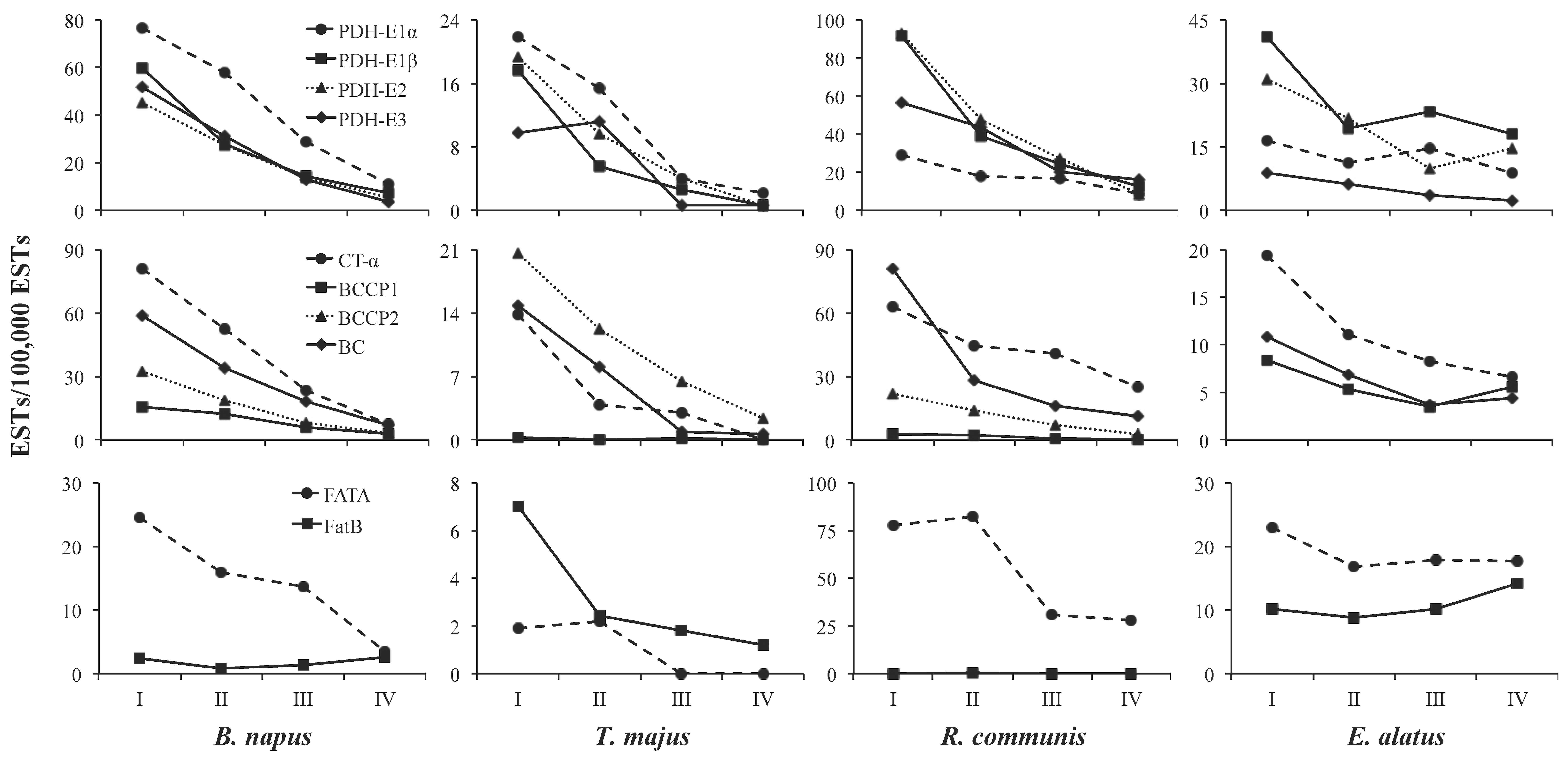

Supplement: Supplementary file 1 [file tpj0068-1014-SD5.tiff]

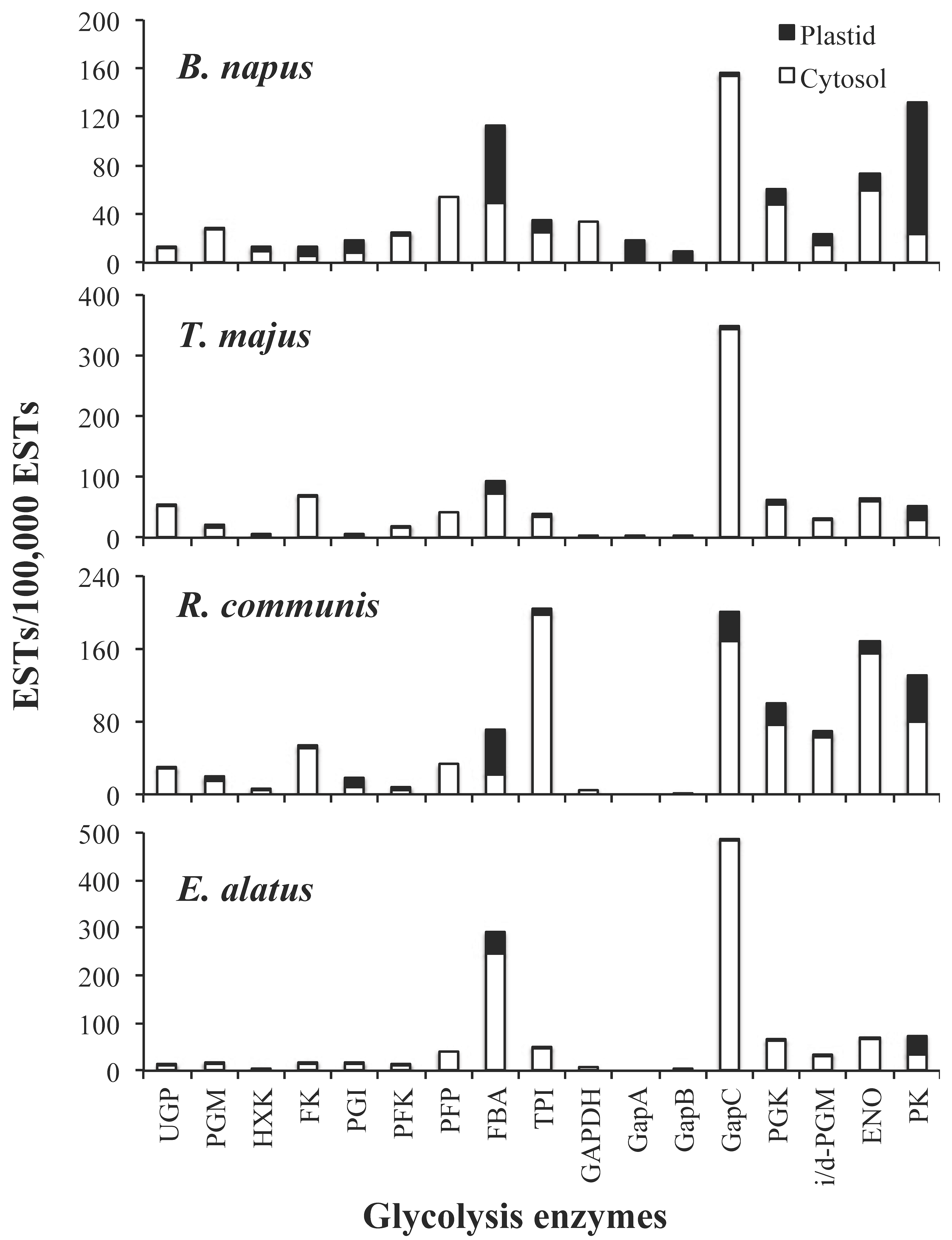

Supplement: Supplementary file 2 [file tpj0068-1014-SD6.tiff]

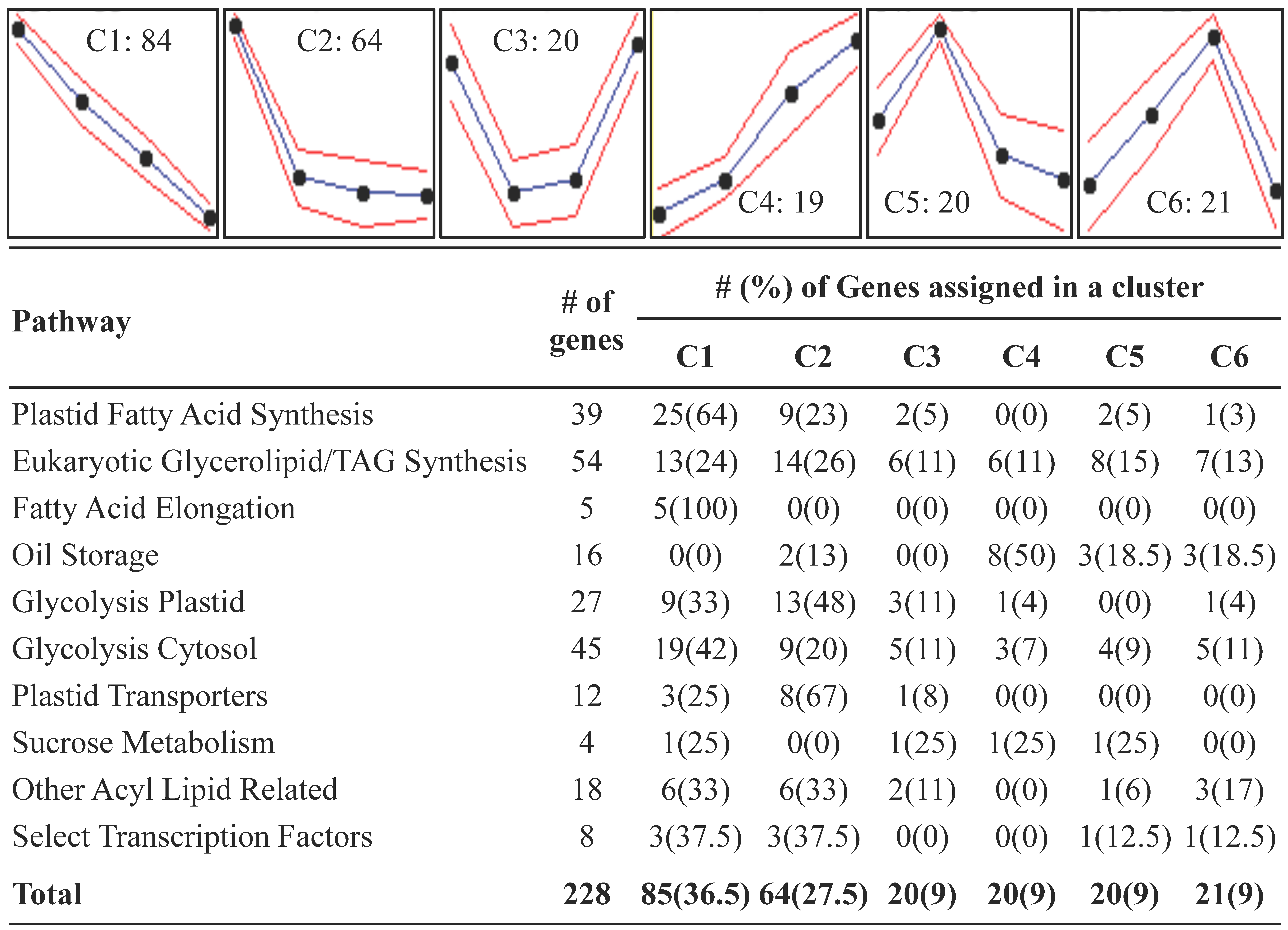

Supplement: Supplementary file 3 [file tpj0068-1014-SD7.tiff]
