# Supplementary material for: Comparative deep transcriptional profiling of four developing oilseeds
Source: Plant J. 2011 Oct 10;68(6):1014–27. doi: 10.1111/j.1365-313X.2011.04751.x (PMC3507003; doi:10.1111/j.1365-313X.2011.04751.x)
Supplement: Supplementary file 4 [file tpj0068-1014-SD1.doc]

**Figure S1.** Temporal profile of EST levels for various plastidial proteins. For abbreviations and annotation, see Table S1a.

**Figure S2.** Comparison of average EST levels between cytosolic versus plastidial glycolytic enzymes in four oilseeds. For enzyme abbreviation and annotation, see Table S1b.

**Figure S3.** Self-organizing maps (SOM) of temporal EST patterns for 228 *B.  napus* proteins of lipid and carbohydrate metabolism. The table indicates the number of total proteins in a pathway that clustered together, with % of genes in parenthesis. Genes included in each pathway are provided in Table S3.

**Table S1a.** Annotation and EST levels for selected genes associated with lipid metabolism. Bold values indicate sum of EST levels for isoforms, subunits, and pathway enzymes. In the columns that indicate ‘avg’, the EST levels of four time points for each species were averaged. To enable comparison of EST levels across stages and between species, data are expressed as ESTs/100 000 ESTs.

**Table S1b.** Annotation and EST levels for selected genes of carbohydrate and organic acid metabolism. Bold values indicate sum of EST levels for isoforms, subunits, and pathway enzymes. In the columns that indicate ‘avg’, the EST levels of four time points for each species were averaged. To enable comparison of EST levels across stages and between species, data are expressed as ESTs/100 000 ESTs.

**Table S2a.** Correlation (*R*_2_) of FAS gene1 expression (average ESTs for four stages) between the species.

**Table S2b.** Correlation (*R*_2_) of FAS gene expression with time, within each species.

**Table S3.** List of genes included in generating self-organized maps for developing embryo of *B.  napus*.

**Table S4.** Details and accession numbers of EST datasets are available on NCBI Short Read Archive (SRA)^1^.

**Table S5a.** Annotation and EST levels for *B.  napus* orthologs of Arabidopsis proteins. The 454 reads obtained for all stages of developing seed were assembled to generate contigs. The contigs were then matched to Arabidopsis TAIR 8 proteins by BLASTX with cutoff *E*-value <E-10. There may be more than one contig/TAIR8 locus ID in which case EST numbers for multiple contigs that matched to the same protein were summed. To enable comparison of EST levels across stages and species, EST counts are expressed as ESTs/100 000 ESTs. In addition to orthologs included in this study (Table S1), all orthologs with ≥10 ESTs are presented. Nucleotide sequence information for the *B.  napus* contigs can be found with corresponding AT locus ID in BnContigSeq.fasta.

**Table S5b.** Annotation and EST levels for *R.  communis* orthologs of Arabidopsis proteins. The 454 reads obtained for all stages of developing seed were assembled to generate contigs. The contigs were then matched to Arabidopsis TAIR 8 proteins by BLASTX with cutoff *E*-value <E-10. There may be more than one contig/TAIR8 locus ID in which case EST numbers for multiple contigs that matched to the same protein were summed. To enable comparison of EST levels across stages and species, EST counts are expressed as ESTs/100 000 ESTs. In addition to orthologs included in this study (Table S1), all orthologs with ≥10 ESTs are presented. Although not discussed in this manuscript, data for *R.  communis* embryos are also provided for comparison. Nucleotide sequence information for the *R.  communis* contigs can be found with corresponding AT locus ID in RcContigSeq.fasta.

**Table S5c.** Annotation and EST levels for *E.  alatus* orthologs of Arabidopsis proteins. The 454 reads obtained for all stages of developing seed were assembled to generate contigs. The contigs were then matched to Arabidopsis TAIR 8 proteins by BLASTX with cutoff *E*-value <E-10. There may be more than one contig/TAIR8 locus ID in which case EST numbers for multiple contigs that matched to the same protein were summed. To enable comparison of EST levels across stages and species, EST counts are expressed as ESTs/100 000 ESTs. In addition to orthologs included in this study (Table S1), all orthologs with ≥10 ESTs are presented. Although not discussed in this manuscript, data for *E.  alatus* embryo and aril are also provided for comparison. Nucleotide sequence information for the *E.  alatus* contigs can be found with corresponding AT locus ID in EaContigSeq.fasta.
